# Suppressing aberrant phospholipase D1 signaling in 3xTg Alzheimer’s disease mouse model promotes synaptic resilience

**DOI:** 10.1038/s41598-019-54974-6

**Published:** 2019-12-04

**Authors:** Krystyn Z. Bourne, Chandramouli Natarajan, Carlos X. Medina Perez, Batbayar Tumurbaatar, Giulio Taglialatela, Balaji Krishnan

**Affiliations:** 1 Mitchell Center for Neurodegenerative Diseases, Department of Neurology, University of Texas Medical Branch, Galveston, Texas 77555 USA; 2 Neuroscience Summer Undergraduate Program, University of Texas Medical Branch, Galveston, Texas 77555 USA

**Keywords:** Cognitive ageing, Neurodegeneration

## Abstract

Current approaches in treatment of Alzheimer’s disease (AD) is focused on early stages of cognitive decline. Identifying therapeutic targets that promote synaptic resilience during early stages may prevent progressive memory deficits by preserving memory mechanisms. We recently reported that the inducible isoform of phospholipase D (PLD1) was significantly increased in synaptosomes from post-mortem AD brains compared to age-matched controls. Using mouse models, we reported that the aberrantly elevated neuronal PLD1 is key for oligomeric amyloid driven synaptic dysfunction and underlying memory deficits. Here, we demonstrate that chronic inhibition using a well-tolerated PLD1 specific small molecule inhibitor is sufficient to prevent the progression of synaptic dysfunction during early stages in the 3xTg-AD mouse model. Firstly, we report prevention of cognitive decline in the inhibitor-treated group using novel object recognition (NOR) and fear conditioning (FC). Secondly, we provide electrophysiological assessment of better synaptic function in the inhibitor-treated group. Lastly, using Golgi staining, we report that preservation of dendritic spine integrity as one of the mechanisms underlying the action of the small molecule inhibitor. Collectively, these studies provide evidence for inhibition of PLD1 as a potential therapeutic strategy in preventing progression of cognitive decline associated with AD and related dementia.

## Introduction

Alzheimer’s Disease (AD) is the most prevalent form of dementia and the sixth leading cause of death. Despite decades of research, there have been relatively poor outcomes of different therapeutic interventions^1,2^. Recent evidence has shifted the focus of therapeutic attention to early stages in the progression of the disease, one of which is synaptic dysfunction^3–10^. Studies from our group^11–13^ and others^14–16^ have reported a role for phospholipase D (PLD) signaling in modulating synaptic function in the brain.

PLD is a lipolytic phosphodiesterase enzyme encoded by a superfamily of genes and is conserved throughout the eukaryotic kingdom^16,17^. The inducible PLD1 and constitutively expressed PLD2 isoforms share 53% homology and have redundant as well as isoform-specific effects on downstream targets. Primarily, both membrane-associated enzymes conduct a transphosphatidylase activity by cleaving the most abundant membrane phospholipid, phosphatidyl choline (PC), into phosphatidic acid (PA) and choline. In doing so, they alter signaling and morphological events affecting synaptic function such as exocytosis, endocytosis, vesicle release and spine dynamics. Moreover, using the protein-protein interaction domains, they play a larger role as signaling partners downstream to membrane-bound targets like tyrosine kinase and G-protein coupled receptors^16,17^.

Our recently published study was the first to demonstrate that aberrant elevated synaptosomal PLD1, not PLD2, is observed in AD hippocampi compared to age-matched controls^18^. Functionally, we reported that this elevated PLD1 plays a key detrimental role in facilitating both (Aβ and tau) oligomer-driven synaptic dysfunction (HFS-LTP) and underlying memory deficit (novel object recognition – NOR). More importantly, we observed that administration^18^ (and also see Suppl. Fig. 1) of a PLD1 specific inhibitor (VU0 155069 or VU01) in the wildtype mice did not change the behavioral response compared to uninjected controls. However, synaptosomal PLD1 levels are elevated following acute administration of toxic oligomers, and inhibition of PLD1 signaling in a pathological state was beneficial in preventing synaptic dysfunction and associated memory deficits^18^.

In the present study, we extend our previous observations by elucidating potential therapeutic intervention using chronic administration of VU01 by exploring the effects in the 3xTg-AD mouse model of AD-like cognitive decline. VU01 is a derivative of halopemide, that was successfully used in the treatment of schizophrenia with minimal side-effects in long-term studies^17,19,20^. Since VU01 administration does not affect wildtype behavior, we addressed the effects of VU01 in preventing synaptic dysfunction (electrophysiology) and the underlying memory deficits (behavior) driven by progressive accumulation of oligomers of Aβ (oAβ) and tau (otau) in 6-month old 3xTg-AD mice using saline-injected age-matched 3xTg-AD siblings where PLD1 levels are elevated^18^ as appropriate controls. Additionally, we observed whether dendritic spine dystrophy is prevented by chronic inhibition of PLD1 as a potential mechanism of action. The preclinical outcomes reported here provide a clear evidence for the therapeutic potential of PLD1 inhibition in preventing progression of cognitive deficits in AD and related dementia.

## Results

### A chronic one-month treatment with PLD1 inhibitor is sufficient to prevent memory deficits in 6-month old 3xTg-AD mice

**Figure 1:**
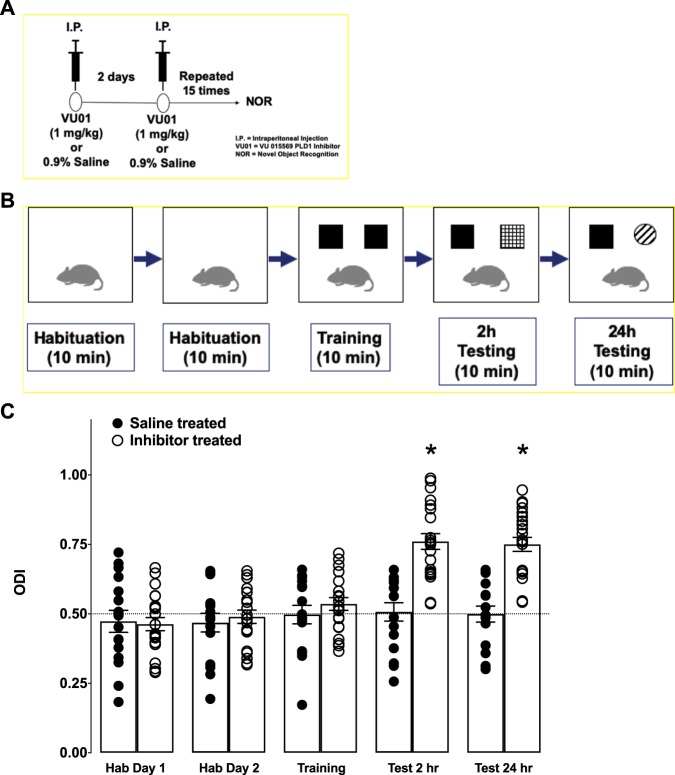
Chronic PLD1 inhibition rescues the NOR deficit in 6-month old 3xTg-AD mice. (**A**) Both female and male 3xTg-AD mice were injected (i.p.) with 1 mg/kg VU01 or 0.9% saline every alternate day to receive a total of 15 injections for a period of one month. (**B**) The schematic depicts the regimen of the protocol described in the methods section. (**C**) Saline treated animals are shown in filled circles while inhibitor treated animals are shown in clear circles. Each dot represents a single animal. The object discrimination index (ODI) measures the relative time spent by the animal in the novel object area. There was no bias observed with any of the areas associated with either objects in both groups of animals during habituation days (since the values are at 0.5 – shown by a dotted line). On the training day, we did not observe any bias for the objects or the associated area for either group suggesting that learning was not affected. There were no differences on any of the trials in the distance travelled, time mobile or time immobile (data not shown) between the groups, providing evidence for the absence of any non-specific effects confounding the experimental measures. There is a significant difference (*p < 0.05, Mann-Whitney U) between the groups at 2 and 24 h test where the inhibitor treated group spent more time with the novel object compared to the saline treated siblings. The saline treated animals do not show any discrimination between the novel object and the familiar object providing evidence for compromised memory.

**Figure 2:**
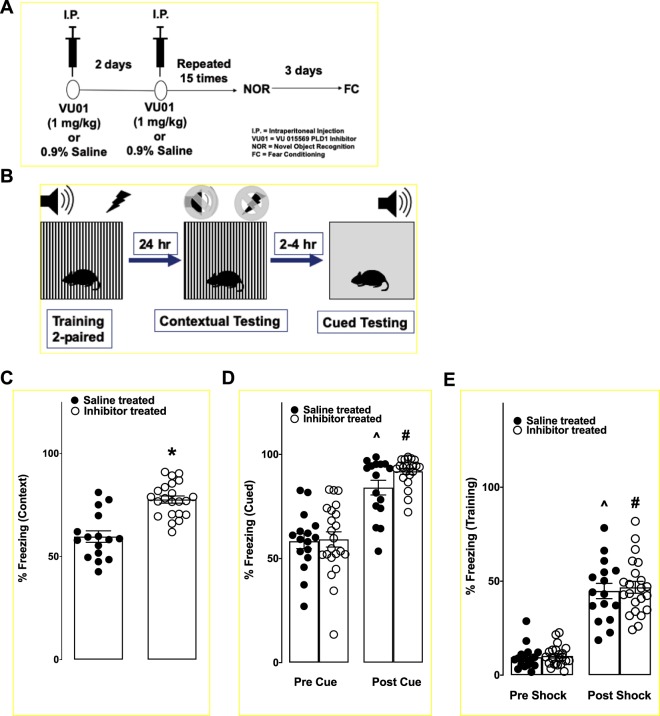
Chronic PLD1 injection prevents the contextual memory deficit in 6-month old 3xTg-AD mice. (**A**) The fear conditioned behavior was conducted in the same group of animals three days following the NOR test; such a sequence strategy allowed for increased handling of the animals. (**B**) Schematic for the fear conditioning training and testing is described in detail in the methods section. After a two-paired training regimen, animals were returned to their cages for 24 h prior to testing in the same context. After contextual testing, the animals were returned to their cages. After 2–4 h, the animals were introduced to a new chamber with different visual, tactile and olfactory characteristics. After a brief pre-cue period where the baseline level of freezing was assessed, the animals were given the sound cue. Once the behaviors were completed, the animals were returned to their cages. (**C**) Saline treated animals are shown with filled circles while the inhibitor treated animals are represented in clear circles. During the contextual phase, the saline treated animals show a greater level of activity (less % freezing) compared to the inhibitor treated group that clustered above 50% freezing, suggesting that more of the inhibitor treated animals had retained the contextual memory associated with the shock. (**D**) In the freezing test, the pre cue (before the sound is played) freezing response is not different between the two groups of 3xTg-AD mice. The post cue response shows a significantly increased freezing response for saline (*^p* < 0.05) and inhibitor (^*#*^*p* < 0.05) compared to their respective pre cue responses. Previous studies of cued memory did report any deficits in the 3xTg-AD^24,25^ mice at 6 months. In agreement with literature, we also report observing no differences in the cued response between the VU01 treated and saline-treated group because of lack of cued memory deficits in the 3xTg-AD mice. (**E**) There were no differences in the two groups of animals in the extent of freezing before and during/after shock. A significantly increased freezing response for saline (*^p* < 0.05) and inhibitor (^*#*^*p* < 0.05) compared to their respective pre shock response was observed. Each point represents one animal.

Male and female transgenic mice were injected i.p. with VU01 (1 mg/kg) (see schematic in Fig. 1A) and subjected to NOR study (Fig. 1B). We observed that the PLD1 inhibitor treated animals showed better NOR compared to their saline treated sibling (*p < 0.05; Mann-Whitney U; Fig. 1C) either at 2 h (saline treated, S: 0.507 ± 0.033 vs inhibitor treated, I: 0.761 ± 0.028) or at 24 h (S: 0.499 ± 0.029 vs I: 0.750 ± 0.026) after training. These results suggest that PLD1 inhibition is effective in preventing detrimental effects induced by toxic oligomers (oAβ and otau) in the perirhinal cortex (implicated in shorter memory retention) as well as the hippocampus (implicated in the longer memory retention)^21^. To further validate our behavioral outcome, we tested the same group of animals using FC (see schematic in Fig. 2A,B), to study hippocampal (contextual) and amygdala (cued)-dependent stronger form of aversive associative memory^22^. While the hippocampal recovery was robustly observed in the PLD1 inhibitor injected mice (S: 59.720 ± 2.798 vs I: 77.670 ± 1.743; *p < 0.05; Mann-Whitney U; Fig. 2C); the cued responses failed to show a significant difference between the two groups (S: 84.040 ± 3.528 vs I: 91.870 ± 1.440; ns; Kruskal-Wallis one-way ANOVA; Fig. 2D). Additionally, the pre-cue freezing was not different between the two groups (S: 58.340 ± 3.585 vs I: 59.180 ± 3.658; ns; Kruskal-Wallis one-way ANOVA; Fig. 2D), but the cued response was significantly different (Fig. 2D; Dunn’s multiple comparison mean ranks) within saline (*^p* < 0.05) and inhibitor-treated mice (^*#*^*p* < 0.05) compared to their respective pre-cued response. Additionally, the two groups did not show any differences in learning the response (pre-shock – S: 9.516 ± 1.635 vs I: 10.220 ± 1.137 & post-shock: 44.780 ± 4.080 vs I: 46.740 ± 3.160; ns; Fig. 2E). We observed significant increase (Fig. 2E; Dunn’s multiple comparison mean ranks) between pre- and post-shock freezing (~ 4-fold increase) in saline (*^p* < 0.05) and inhibitor-treated mice (^*#*^*p* < 0.05). Previous studies (including our own on Tg2576^23^ and others on 3xTg-AD^24,25^) of mouse models of AD-like memory deficits routinely report that cued memory response is intact while contextual memory response is compromised, presumably due to differences in mice anxiety specific confounds. Thus, we corroborate here that cued freezing memory is intact in the saline-treated 3xTg-AD group (as reported in earlier studies) and the inhibitor treatment does not modify the cued memory response. Therefore, we report a preservation of contextual (hippocampal) memory by chronic PLD1 inhibition in 6-month 3xTg-AD mouse model.

### VU01-treated 6-month old 3xTg-AD mice shows improved synaptic function compared to saline-treated age-matched sibling group

**Figure 3:**
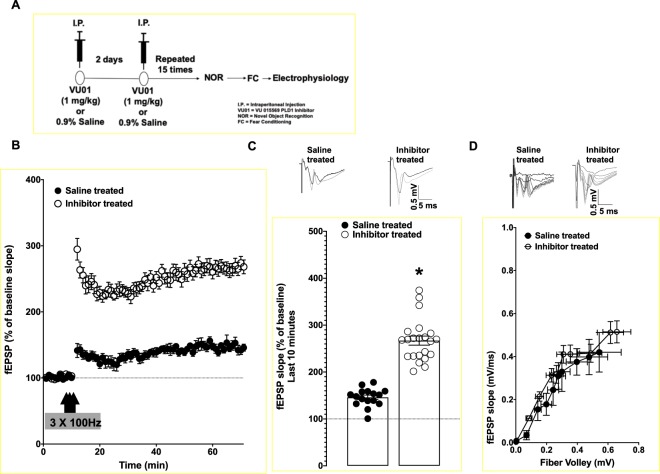
High Frequency Stimulation associated long term potentiation in the Schaffer collateral synapses is preserved in the hippocampi of chronic PLD1 inhibitor treated 6-month old 3xTg-AD mice. (**A**) The group of animals subjected to the behaviors were assessed after the last day of FC test (minimum one day) for the electrophysiology studies. (**B**) Inhibitor-treated group (clear circles) showed increased potentiation compared to saline treated sibling group (filled circles) following high frequency stimulation (HFS – 3 × 100 Hz). (**C**) Long-term potentiation (LTP) measured for the last 10 minutes of the recording shows a significant difference (*p < 0.05) in the inhibitor treated group (clear circles) compared to the saline treated group (filled circles). Each dot represents the number of animals used. Representative traces for pre- (black trace) and post-HFS (grey trace) are provided. (**D**) The basal synaptic strength measured using the fiber volley vs the field excitatory post-synaptic potential is plotted for the saline treated (filled circles) and the inhibitor treated group (clear circles). There was no significant difference observed between the two groups. Representative traces for each group are provided alongside.

Following behavioral assessments (see schematic in Fig. 3A), we addressed whether the prevention of cognitive decline in PLD1 inhibitor treated animals could be observed at the synaptic level. In our previous study, we demonstrated a role for oligomer-driven PLD1 dependent synaptic dysfunction that resulted in reduced high frequency stimulation dependent long term potentiation (HFS-LTP) in the Schaffer collateral hippocampal synaptic pathway^18^. As a result, we assessed potentiation and found that 3xTg-AD mice that received chronic PLD1 inhibitor treatment showed significantly elevated HFS-LTP (*p < 0.05; Mann-Whitney U; Fig. 3B,C) compared to saline-treated siblings (Fig. 3C; Last 10 min responses - I: 267.000 ± 9.631 vs S: 146.100 ± 4.809 respectively), suggesting that the preservation of contextual memory seen in NOR and FC could be a consequence of preservation of synaptic function in the Schaffer collaterals. We did not observe any differences in the synaptic strength (Fig. 3D) between the two groups of mice.

### Dendritic spine morphology is better maintained following chronic PLD1 inhibition in 6-month old 3xTg-AD mice compared to saline-treated controls

**Figure 4:**
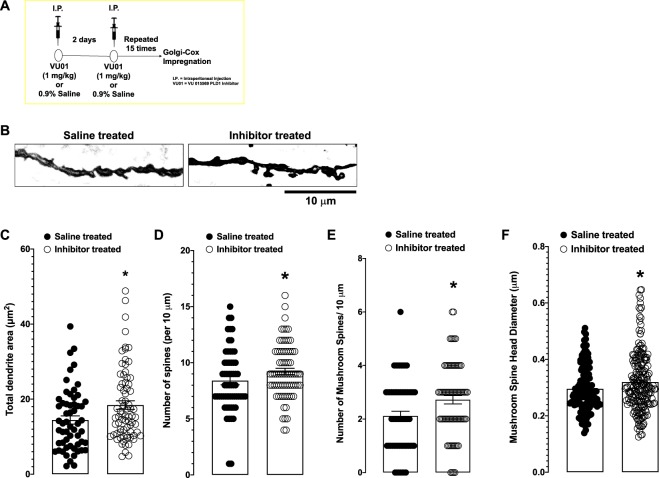
Chronic PLD1 inhibition in 6-month old 3xTg-AD mouse model of AD-like cognitive decline provides synaptic resilience by preserving dendritic spine integrity. (**A**) A separate cohort of 3xTg-AD mice were injected using the schematic described and the brains removed immediately after the last injection and processed for Golgi-Cox impregnation as described in the methods section. (**B**) Representative dendrites from saline and inhibitor treated groups. (**C**) The total dendritic area was significantly (*p < 0.05) increased in the inhibitor treated (clear circles) compared to the saline treated (filled circles) group. (**D**) The total number of spines per 10 μm of the selected dendrites (using the criteria described in the methods section) were significantly greater (*p < 0.05) in the inhibitor treated (clear circles) compared to the saline treated group (filled circles). The number of filamentous or thin spines and stubby spines per 10 μm did not reach significance in the inhibitor treated compared to the saline treated group (data not shown). (**E**) The number of mushroom spines per 10 μm, however, were significantly greater (*p < 0.05) in the inhibitor treated (clear circles) compared to saline treated group. (**F**) In addition, there were significantly (*p < 0.05) greater numbers of mushroom spines that had a larger diameter in the inhibitor treated (clear circles) compared to saline treated group (filled circles), suggesting that the inhibitor prevented the loss of mushroom spines, particularly the ones with larger head diameters that are considered to be fully functional in memory mechanisms.

During development, PLD1 plays an important role in regulating dendritic spine morphology and integrity^26–28^. Since we observed improved synaptic function in VU01-treated group (Fig. 3), we further explored whether synaptic function maintenance is observed in the morphology of dendritic spines in the Schaffer collateral pathway. We used the well-established and optimized Golgi-Cox impregnation technique^29^, because of its ability to analyze spine morphology through visualization of a low percentage of neurons (see schematic in Fig. 4A). We observed a significant difference (*p < 0.05; Mann-Whitney U) in the dendritic area (S: 14.450 ± 1.090 vs I: 18.420 ± 1.129, Fig. 4C); number of dendrites per 10 μm (S: 8.400 ± 0.364 vs I: 9.227 ± 0.281, Fig. 4D) and number of mushroom spines per 10 μm (S: 2.117 ± 0.175 vs I: 2.720 ± 0.157, Fig. 4E) in the CA1 region of the inhibitor treated compared to saline treated group. In addition, the mushroom spine head diameter in VU01 treated group was significantly greater than the saline treated group (S: 0.296 ± 0.007 vs I: 0.320 ± 0.007). We did not observe significant differences (data not shown) in other parameters such as dendrite diameter μm (S: 0.334 ± 0.0156 vs I: 0.363 ± 0.015), the number of filamentous (S: 3.175 ± 0.199 vs I: 3.389 ± 0.190) or stubby spines (S: 3.271 ± 0.260 vs I: 3.342 ± 0.209) per 10 μm.

## Discussion

Synapse loss is one of the early events in the progressive cognitive decline^30^. Importantly, progressive accumulation of low molecular weight aggregates of the amyloidogenic proteins, Aβ (oAβ) and tau (otau), can drive synaptic dysfunction and memory deficits leading to synaptic loss. Thus, there is an urgent need to identify downstream effectors and aberrant signaling events underlying progressive synapse dysfunction that are amenable to therapeutics.

In the present study, we report that chronic inhibition with a PLD1 specific inhibitor (VU 0155069) is sufficient to prevent the cognitive deficits driven by progressive accumulation of oAβ and otau in the 6-month old 3xTg-AD mice, by (1) studying behavior (Figs. 1 and 2) to assess prevention of memory deficits, (2) electrophysiology (Fig. 3) looking at the prevention of synaptic dysfunction and finally (3) elucidating potential synaptic resilience via preservation of dendritic spines (Fig. 4).

Overexpression of PLD activity in AD brains^31^ was known for decades, well before the mammalian isoforms were discovered. Phosphatidyl choline (PC), the most abundant membrane phospholipid, is the major substrate for PLD1 and PLD2. To normalize the PLD activity, PC administration was increased (in the form of lecithin) among AD patients. The supplementation showed improvement in cognitive function for a few days^32^. However, this PLD substrate alone failed to prevent the cognitive decline, suggesting that inhibiting PLD expression/function should be explored as a better therapeutic strategy^32^.

However, previous studies on PLD isoforms were unclear in elucidating the specific isoform to be targeted for therapeutics. A study investigating mouse hippocampal mossy fiber sprouting reported increased expression of both PLD1 and PLD2 isoforms^33^. Immunohistochemistry localized PLD1 in the neurons while PLD2 was found in the glia. While other studies of PLD isoforms in pathological states of AD^34,35^ post-mortem brains and a rodent model of scrapie^36^ reported elevated PLD1 expression in enriched membrane fractions. But they concluded that the PLD1 expression was localized to mitochondria and glia without reporting on PLD2 expression (53% homology to PLD1 that was already known to be localized to glia). Likewise, there is a unique mitochondrial PLD (PLD6) linked to the outer membrane with N-terminal tail that has homology to PLD1 and PLD2 that may have been the mitochondrial PLD reported in this study, but not verified by additional approaches^16^. Interestingly, another set of cellular studies proposed a neuroprotective role for PLD1, where it decreased γ-secretase activity, thereby reducing Aβ formation^37^ and promoted trafficking of βAPP^38^, thereby clearing it. But there were no mouse model studies that verified this protective role by overexpressing PLD1 in a pathological state. Another group studied only PLD2 specific effects using double-transgenic mouse model of human Aβ overexpression (Tg2576) crossed with PLD2 knockout^39^. While ablating PLD2 expression suppressed Aβ-driven synaptic and behavioral dysfunction, the study did not corroborate their findings (validating PLD2 overexpression) in human clinical samples or report on whether PLD2 is important for tau-related synaptic events. Neuropathology of tau is reported in 22 different neurodegenerative states, including AD, with distinct effects on synaptic function^40^. It is also important to note here that PLD isoforms are also abnormally elevated or aberrantly recruited in signaling leading to other pathological states, notably cancer^16^. As a result, there was a lot of interest in developing well-tolerated specific small molecule inhibitors for each isoform^17,19,41^. Thus, there was enough evidence to pursue PLD isoforms as potential therapeutic targets in AD and related dementia. However, there was a pre-requisite to establish (1) which isoform(s) is(are) overexpressed in human pathological states of AD and (2) whether the isoform is downstream to synaptic events underlying both known drivers (oAβ and otau) of synaptic dysfunction and underlying memory deficits.

In response, our previous study conducted a systematic approach and identified that PLD1 (not PLD2) is overexpressed in the crude synaptosomal fractions isolated from post-mortem AD brain hippocampi compared to age-matched controls^18^. More importantly, we observed that synaptosomal PLD1 was progressively elevated in the 3xTg-AD mouse model with age. This was encouraging since the synaptic dysfunction and associated memory deficits in this transgenic mouse model overexpressing the human genes for amyloid precursor protein, presenilin and tau was modeling the human pathological condition. As a result, we next addressed whether PLD1 was, as speculated in cellular studies, neuroprotective against Aβ^37,38^. We determined the role of PLD1 by using our well-characterized PLD1 specific small molecule inhibitor, VU 0155069 (also called VU01). We hypothesized that PLD1, if neuroprotective, when inhibited will exacerbate the synaptic dysfunction driven by oAβ or otau. Surprisingly, we observed the exact opposite, PLD1 inhibition blocked the HFS-LTP deficit driven by either oAβ or otau^18^. Interestingly, we also validated that PLD2 specific inhibition was protective against the synaptic insults of oAβ, but was ineffective against the otau-driven HFS-LTP deficits. Thus, we speculated that PLD1, the inducible isoform, that is responsive to many upstream regulators is recruited by both oAβ and otau, thereby enhancing its therapeutic relevance. In the same study, we also verified that acute PLD1 inhibition was effective in attenuating synaptic dysfunction observed in 3xTg-AD mice, while clearly noting that inhibiting PLD1 does not affect NOR memory responses in wildtype mice.

Synaptic dysfunction and cognitive deficits in the 3xTg-AD mouse model are driven by overexpression of human genes that progressively increase oAβ and otau levels^42,43^. In order to assess the therapeutic potential, we rationalized that the effect of PLD1 inhibition can be effectively compared only in the pathological state and as a result, we used age-matched 3xTg-AD siblings injected with saline as the control group for the PLD1 inhibitor injected experimental group in the current studies, since PLD1 inhibition in wildtype mice does not affect memory^18^ (also see Suppl. Fig. 1). At 6 months of age, the 3xTg-AD mice show synaptic dysfunction as well as memory deficits in conjunction with detectable levels of both oligomers^42,43^. Thus, we applied pharmacological PLD1 specific inhibitor at one log scale less than our published acute administration of 10 mg/kg, but sustained the low-level inhibition using repeated injections over a month beginning at 5 months of age. At the current regimen of 15 injections of 1 mg/kg of VU01 over a month, there were no overt morphological deficits that we observed in the VU01 injected group which is in keeping with the literature that purports the well-tolerated minimal side-effect profile^19,20^.

For all our studies, we tracked the gender as one of the factors between the different experimental procedures, since previous studies had reported variations between the genders^44^. The animals that were used in this study were bred in UTMB and were the progeny of outbred 3xTg-AD mice using the wildtype backgrounds (C57Bl/6 and 129SV) from which the transgenic line was originally generated. The genotypes were validated using RT-PCR and western blots (Batbayaar Tumurbaatar, personal communication). In our current experiments, we did not observe significant differences between male and female mice within the same experimental group and therefore, we pooled the results.

We observed that such PLD1 inhibition applied during the early stages in the progression of oAβ and otau is beneficial in preventing both the perirhinal cortex dependent shorter retention (2 h NOR test, Fig. 1C) and the hippocampal dependent (24 h NOR, Fig. 1C and 24 h contextual FC, Fig. 2C) deficits observed in age-matched saline injected siblings. The reproducibility of hippocampal specific rescue was verified at the Schaffer collateral synapses (Fig. 3), where there is a further 100% increase in the potentiation of the glutamate neurotransmission rich CA1 synaptic response. First, we explored the dendritic spine characteristics in the CA1 region (Fig. 4). Recently, dendritic spine preservation^45^ was reported to be a mechanism that contributes to synaptic resilience observed among a group of individuals aged 80 and above that show cognitively normal status despite levels of Aβ plaques and neurofibrillary tangles that meet National Institute of Aging-Reagan criteria of intermediate to high likelihood of AD^46–48^. During development, PLD1 modulates dendritic spines by inhibiting dendritic spine branching and increasing dendritic spine morphogenesis in hippocampal primary neurons^26–28^. But, post-developmental overexpression, particularly in a pathological state (extensively explored in cancer progression)^16,17^, is reported to result in aberrant downstream signaling that enhance the diseased states. Therefore, we hypothesized that oAβ and otau recruitment of post-developmental elevated PLD1 could be detrimental instead of beneficial for hippocampal spines. Thus, inhibiting the action of progressive oAβ and otau by preventing downstream PLD1 function could be reflected in better preservation of the dendritic spine morphology. Indeed, we observed an increased number of large mushroom spines (Fig. 4) in the inhibitor treated group, suggesting that preventing PLD1 overexpression promotes resilience to mushroom spine loss, well-documented in progression of AD^45^. We are currently expanding on other possible mechanisms that account for the synaptic plasticity and cognition effects of VU01 chronic administration. One such approach involves looking at dendritic dystrophy prevention via prevention of filamentous actin depolymerization^49^. We are currently exploring mechanisms such as mechanical target of rapamycin (mTOR) and protein kinase C alpha (PKCα) dependent changes of cofilin phosphorylation and its effects on filamentous acting depolymerization as one of the possible mechanisms maintaining the synaptic integrity by preserving the dendritic spines. Additional possibilities include autophagy and neuroinflammation that we plan to explore in future studies towards explaining the role of chronic VU01-based preservation of synaptic function and underlying memory that we observed.

It is important to note here that despite their essential roles, single^16,39,50^ or double^51^ transgenic PLD isoform knockouts do not cause lethality, suggesting that there is inbuilt redundancy for survival. This increases the enthusiasm in the potential therapeutic application of PLD1 inhibitor in preventing AD-related cognitive decline. Finally, early diagnosis of synapse loss is possible using synaptic vesicle glycoprotein 2 A (SV2A) in positron emission tomography (PET) in living brain^52^. SV2A as a potential biomarker is being explored. If successful, then such early synapse loss can be targeted by repeated administration of PLD1 inhibitor as a preventative. The improved synaptic resilience that results by protection against dendritic spine loss may be an effective therapeutic in preventing the progression of the cognitive decline in AD and related dementia.

## Materials and Methods

### Drugs

PLD1 inhibitor (VU0 155069) was obtained from Tocris Bioscience (Bio-Techne, Minneapolis, NE).

### Animals

Male and female 3xTg-AD transgenic mice were purchased from Jackson Labs (Bar Harbor, ME) and maintained through a breeding program at UTMB. Male C57Bl/6 mice (n = 61) were also purchased from Jackson Labs for conducting the wildtype study described in Suppl. Fig. 1. Mice were housed upto five per cage in their filter-top cages in a temperature-controlled environment at 22 °C, humidity 40%, and a 12:12 h light–dark cycle, with regular chow provided *ad libitum*. We had to utilize three cohorts of the 3xTg-AD mice to get a minimum of 15 animals [Round 1: 13 females (VU01 injected) and 2 females (0.9% saline); Round 2: 2 females & 7 males (VU01 injected) and 7 females & males each (0.9% saline); Round 3: 2 females & 3 males (VU01 injected) and 2 females & 2 males (saline injected)], separated by few months to complete the experiments described here. Thus, we had n = 16 animals total for saline injected and n = 22 animals total for VU01 injected to conduct the behavioral and electrophysiological studies. In addition, we had n = 5 animals for VU01 injected and n = 4 for saline injected to conduct the Golgi experiments described here. Animals received a single injection intraperitoneally (i.p.) of 1 mg/kg of VU01 diluted in 0.9% saline solution (inhibitor treated) or an equivalent amount of 0.9% saline (saline treated) and returned to their cage. Based on previous reports, we analyzed all our results separately, but did not find significant difference between the sexes for any of our assessments at this age. As a result, we have represented the combined results for this study. The study was approved by the Institutional Animal Care and Use Committee (IACUC) of the University of Texas Medical Branch (UTMB, Galveston, TX, USA) and was performed National Institutes of Health Guidelines^53^ on the use of laboratory animals. All methods and experiments were performed in accordance with the UTMB and NIH approved relevant guidelines and regulations. All behavioral testing was done within the 12 h light cycle for return to home cages prior to the 12 h dark cycle. After completion of the behaviors for the first two cohorts, animals were processed as described under field electrophysiological recordings section. The last cohort of animals for Golgi staining were also deeply anesthetized with isoflurane, and immediately the brain was extracted from the skull, washed with phosphate buffered saline pH 7.4 (ThermoFisher Scientific, Waltham, MA) and processed as described under Tissue Processing and Golgi Staining section.

### Field electrophysiological recordings

Our standard protocol was used as previously described^18,54–57^. Briefly, mice were deeply anesthetized with isoflurane and transcardially perfused with ~30 mL of room temperature carbogenated (95% O_2_ and 5% CO_2_ gas mixture) NMDG-artificial cerebrospinal fluid (aCSF) (in mM 93 NMDG, 2.5 KCl, 1.2 NaH_2_PO_4_, 30 NaHCO_3_, 20 C_8_H_18_N_2_O_4_S, 25 C_6_H_12_O_6_, 5 C_6_H_7_O_6_Na, 2 CH_4_N_2_S, 3 C_3_H_3_NaO_3_, 10 MgSO_4_,7H_2_0, 0.5 CaCl_2_,2H_2_O, 12 C_5_H_9_NO_3_S, pH 7.4) and sliced using Compresstome VF-300 (Precisionary Instruments, Greenville, NC) in carbogenated NMDG-aCSF to obtain 350 μm transverse brain sections. Slices were allowed to recover for 10 min in carbogenated NMDG-aCSF at 33 °C. Slices were then maintained at room temperature in a modified carbogenated HEPES holding aCSF solution (in mM 92 NaCl, 2.5 KCl, 1.2 NaH_2_PO_4_, 30 NaHCO_3_, 20 C_8_H_18_N_2_O_4_S, 25 C_6_H_12_O_6_, 5 C_6_H_7_O_6_Na, 2 CH_4_N_2_S, 3 C_3_H_3_NaO_3_, 2  MgSO_4_,7H_2_0, 2 CaCl_2_,2H_2_0, 12 C_5_H_9_NO_3_S, pH 7.4). Slices were recorded in carbogenated standard recording naCSF (in mM 124 NaCl, 2.5 KCl, 1.2 NaH_2_PO_4_, 24 NaHCO_3_, 5 C_8_H_18_N_2_O_4_S, 13 C_6_H_12_O_6_, 2 MgSO_4_,7H_2_0, 2 CaCl_2_,2H_2_0, pH 7.4). Evoked field excitatory post-synaptic potentials (fEPSPs) recordings were performed by stimulating the Schaffer collateral pathway (located in stratum radiatum) using a stimulating electrode of ~22 kΩ resistance placed in the CA3 region and glass recording electrodes in the CA1 region. Current stimulation was delivered through a digital stimulus isolation amplifier (A.M.P.I, ISRAEL) and set to elicit a fEPSP approximately 30% of maximum for synaptic potentiation experiments using platinum-iridium tipped concentric bipolar stimulating electrodes (FHC Inc., Bowdoin, ME). The use of platinum iridium wire and diphasic pulses can help minimize electrode polarization^58^. Using a horizontal P-97 Flaming/Brown Micropipette puller (Sutter Instruments, Novato, CA), borosilicate glass capillaries were used to pull recording electrodes and filled with naCSF to get a resistance of 1–2 MΩ. Field potentials were recorded in CA1 stratum radiatum using a Ag/AgCl wire in CV7B headstage (Molecular Devices, Sunnyvale, CA) located ~1–2 mm from the stimulating electrode. LTP was induced using a high frequency stimulation protocol (3 × 100 Hz, 20 seconds) as previously described^18,54–57^. Recordings were digitized with Digidata 1550B (Molecular Devices, Sunnyvale, CA), amplified 100X and digitized at 6 kHz using an Axon MultiClamp 700B differential amplifier (Molecular Devices) and analyzed using Clampex 10.7 software (Molecular Devices). To assess basal synaptic strength, 250 μs stimulus pulses were given at 10 intensity levels (range, 100–1000 μA) at a rate of 0.1 Hz. Three field potentials at each level were averaged, and measurements of fiber volley (FV) amplitude (in millivolts) and fEPSP slope (millivolts per millisecond) were performed using Clampfit 10.7 software. Synaptic strength curves were constructed by plotting fEPSP slope values against FV amplitudes for each stimulus level. Baseline recordings were obtained for 10 min by delivering single pulse stimulations at 20 second intervals. All data are represented as a percentage change from the initial average baseline fEPSP slope obtained for the 10 min prior to HFS. Two slices were recorded per animals and were averaged to provide the data as number of animals per group.

### Behaviors

#### Novel object recognition

NOR was performed as described previously^18,54,55,57^. Briefly, animals were habituated for two consecutive days and assessed for normal locomotion and acclimation to the test environment (see schematic in Fig. 2A). After placement in the open field box for two 10-min test sessions that are 24 h apart, the Any-maze (Stoelting Co., Wood Dale, IL) video tracking software quantifies various locomotor parameters: total distance traveled, time spent moving >50 mm/sec, number of rears, number of entries into and time spent in the center 1/9th of the locomotor arena. Twenty-four hours after the last habituation session, animals were subjected to training in a 10 min session of exposure to two identical, non-toxic objects (hard plastic items) in the open field box. The time spent exploring each object was recorded using an area 2 cm^2^ surrounding the object and was defined such that nose entries within 2 cm of the object was recorded as time exploring the object. After the training session, the animal was returned to its home cage. After a retention interval of 2 h and subsequently 24 h, the animal was returned to the arena in which two objects, one identical to the familiar object but previously unused (to prevent olfactory cues and prevent the necessity to wash objects during experimentation) and one novel object. The animal was allowed to explore for 10 min, during which the amount of time exploring each object was recorded. Objects were randomized and counterbalanced across animals. The animals were returned to their home cages with food/water ad-libitum for 24 h minimum. After the rest period of minimum three days, the animals were tested for fear conditioning as described below. For novel object recognition tests, the percent time exploring each object (familiar versus novel) is reported as an object discrimination index (ODI). An index above 0.5 is indicative of novelty associated with the object. Each mouse was tested at 2 h and at 24 h with the intention of assessing the shorter and longer time frames in memory recall. Different novel objects (color and shape) were used in the 24 h test compared to the 2 h test, to avoid performance deficits.

#### Fear conditioned response

Contextual and cued fear conditioned responses were assessed using our standard two‐pairings fear conditioning training protocol as previously described^23^, utilizing the UTMB Rodent *In Vivo* Assessment Core Facility. Briefly, the standard protocol consisted of a training phase when the mice were placed in a particular environment - a standard mouse fear conditioning chamber (Med Associates, Fairfax, VT, a training chamber with particular lighting, geometry, odor that constitutes the context conditioned stimulus, CS) and allowed to explore for 3 min. An auditory CS (80 dB white noise) was then presented for 30 s and one footshock (0.8 mA, 2 s duration; the unconditioned stimulus, US) delivered during the last 2 s of the auditory CS. A second presentation of the auditory CS and the US was delivered at the 5 min mark and the animals then left in the cage for another 2 min. Twenty-four hours later, the mice were returned to the same training chamber and the context test for fear learning performed. The amount of freezing the mice exhibited during 5 min in the training chamber was measured. Between two and four hours later, the cued test was performed in a completely novel context. The animals were placed in the testing chamber and freezing was measured for 3 min before the auditory CS was represented and freezing quantified over the next 3 min. Freezing was quantified using FreezeFrame automated video capture and software analysis (Coulbourn Instruments, Whitehall, PA, USA) and evaluated as percentage freezing in 30 s (training) or 60 s (contextual, cued) bins. Epochs were averaged to provide the data as number of animals per group.

### Tissue processing and Golgi–Cox staining

Brain hemispheres, obtained as described in the animals sections, were stained using the FD Rapid Golgi Stain Kit (PK401, FD Neurotechnologies, Columbia, MD) and the manufacturer’s instructions. Tissue slices were impregnated in chromate mixture of Solution A (potassium dichromate and mercuric chloride) and Solution B (potassium chromate). The chromate solution was replaced after the first 24 h, and tissue was then left in chromate solution in the dark for 15 days. Next, tissue slices were immersed in Solution C for 24 h, and this solution was replaced after 24 h, according to manufacturer’s instructions. These brains were shipped to FD Neurotechnologies, where they were sliced in 30 μm sections, mounted three per slide on gelatin coated slides, sequentially for each animal. All the slides were then processed by the manufacturer, mounted and shipped back for further microscopic assessments. Slides were stored in darkness.

### Dendrite imaging

Previously published criteria and standards for dendritic imaging were used^45,59^. All imaging and subsequent analysis was conducted by a single, blinded experimenter. Golgi stained neurons were imaged at high magnification (100X oil-immersion objective) using the brightfield options in the All-in-One Fluorescence Microscope BZ-X800E (Keyence Corporation of America, Itasca, IL). Images were magnified further using a 3X optical zoom so that the morphology of individual spines could be determined and subsequently quantified. Z-stack images were collected at 0.3 μm intervals to cover the full depth of the dendritic arbors (20–30 μm) and then compressed into a single TIFF image using the BZ-H4A software. Subsequent quantitative analysis used the ImageJ software (Open Source from National Institutes of Health, Bethesda, MD) on these TIFF stacks. For each animal, one slide containing 3 hemispheric slices with the best representation of the Schaffer collateral was chosen. From each tissue slice, 5 cells were imaged and analyzed. The following criteria were used to select cells for imaging: (1) located centrally within the tissue sample depth, (2) not obscured by large staining debris, and (3) fully impregnated. If the cell met the criteria, a single dendritic length was imaged. Dendrite selection criteria were: (1) unobstructed/isolated/not overlapping other dendrites, (2) length >30 µm, and (3) diameter approximately 1 µm. If >2 dendrites fulfilled the criteria from a single cell, the first dendrite clockwise was the only dendrite selected. Each tissue slice was initially imaged under low 20X magnification to establish the region of interest.

### Statistics

All data are reported as mean ± SEM. Statistical significance was calculated using GraphPad Prism 8 (San Diego, CA). All statistical tests were 2‐tailed, with the threshold for statistical significance set at 0.05. To account for non-normal distribution of data, either non-parametric t-tests (Mann-Whitney U or Wilcoxon rank sum) or one-way ANOVA (Kruskal-Wallis test) followed by Dunn’s multiple comparison when significance was achieved, were used.

## Supplementary information

VU01 concentration response in wild type mice fear conditioning behavior

## Data Availability

The datasets used and generated in this study are available from the corresponding author upon reasonable request.
