# Supplementary material for: Suppressing aberrant phospholipase D1 signaling in 3xTg Alzheimer’s disease mouse model promotes synaptic resilience
Source: Sci Rep. 2019 Dec 4;9:18342. doi: 10.1038/s41598-019-54974-6 (PMC6892889; doi:10.1038/s41598-019-54974-6)
Supplement: Supplementary file 1 — VU01 concentration response in wild type mice fear conditioning behavior [file 41598_2019_54974_MOESM1_ESM.docx]

Supplementary Information for

**Suppressing aberrant phospholipase D1 signaling in 3xTg Alzheimer’s disease mouse model promotes synaptic resilience**

**Authors:** Krystyn Z. Bourne^1^, Chandramouli Natarajan^1^, Carlos X. Medina Perez^2^, Batbayar Tumurbaatar^1^, Giulio Taglialatela^1^ and Balaji Krishnan^1^

^1^Mitchell Center for Neurodegenerative Diseases, Department of Neurology, University of Texas Medical Branch, Galveston, Texas, USA, 77555

^2^Neuroscience Summer Undergraduate Program, University of Texas Medical Branch, Galveston, Texas, USA, 77555

***Corresponding Author:** Balaji Krishnan. Email: bakrishn@utmb.edu. Address: 301 University Blvd. Galveston, TX 77555-1045. Phone No: (409) 772-8069. Fax No. (409) 772-0015

(464 words) Supplementary Figure 1: PLD1 inhibition does not alter fear conditioned behavior in 3-month old C57Bl/6 mice. A) Male 3xTg-AD mice were injected (i.p.) with either none (dummy – just needle poke) or 1, 3, 10, 30 mg/kg VU01 or vehicle alone (0.9% saline) one hour before conducting fear conditioning studies. We used n=6 mice for the dummy injections, while 11 mice were used for each of the other experimental groups. B) The schematic depicts the regimen of the protocol, also described in the methods section. In all the graphs (C, D, E), the following color schematic has been used - Dummy (D, black), Vehicle or Saline (S, blue), 1 mg/kg (1, brown), 3 mg/kg (3, green), 10 mg/kg (10, dark blue), 30 mg/kg (30, pink). Each dot represents a single animal. C) In the contextual memory test, all groups demonstrated robust freezing response to the environment used for the shock training (D: 62.540 ± 1.808; S: 72.200 ± 3.542; 1: 73.240 ± 2.350; 3: 68.140 ± 3.634; 10: 65.850 ± 5.188; 30: 73.600 ± 3.381), but there was no significant difference between the groups. D) In the cued memory test, all groups showed minimal freezing to the new environment (pre-cue depicted in clear circles - D: 1.235 ± 0.515; S: 0.497 ± 0.265; 1: 2.055 ± 1.260; 3: 3.272 ± 1.757; 10: 3.196 ± 1.573; 30: 3.566 ± 1.050), with no significant difference between any of the groups. Upon sound cue, (post-cue depicted in filled circles – D: 64.010 ± 5.221; S: 54.960 ± 9.674; 1: 68.010 ± 4.642; 3: 61.470 ± 7.377; 10: 61.810 ± 8.404; 30: 71.350 ± 6.830), all groups showed significantly greater freezing compared to their respective pre-cue responses (**p*<0.05; Kruskal-Wallis one-way ANOVA). However, the post-cued memory at any of the inhibitor concentrations was not significantly different from the saline or dummy-treated group. E) During training, all groups showed minimal freezing response to the environment before the shock (pre-shock depicted in clear circles – D: 1.383 ± 0.570; S: 2.148 ± 0.516; 1: 2.010 ± 0.864; 3: 2.567 ± 0.674; 10: 2.821 ± 0.625; 30: 4.884 ± 0.977). While the post-shock (depicted in filled circles: D: 15.200 ± 4.058; S: 16.060 ± 3.771; 1: 15.140 ± 3.322; 3: 19.370 ± 3.223; 10: 14.600 ± 2.685; 30: 23.75 ± 3.588) were significantly different (**p*<0.05; Kruskal-Wallis one-way ANOVA) compared to their respective pre-shock responses, there was no significant difference between the groups in extent of post-shock freezing. Collectively, these observations provide a clear indication that the PLD inhibitor does not affect behavioral responses to fear conditioned memory or novel object memory (see our recently published study^18^). As a result, we decided to use age-matched 3xTg-AD siblings injected with saline as the appropriate controls to test the therapeutic potential of PLD1 inhibitor in the current study.
